# Assessment of inter-individual variability in hamstring muscle recovery after a sport-specific sprint training in women and men

**DOI:** 10.3389/fphys.2023.1331878

**Published:** 2024-01-09

**Authors:** Pedro L. Cosio, Lia Moreno-Simonet, Aniello Porcelli, Mario Lloret, Xavier Padulles, Josep M. Padulles, Andreu Farran-Codina, Joan A. Cadefau

**Affiliations:** ^1^ Institut Nacional d’Educació Física de Catalunya (INEFC), Universitat de Barcelona (UB), Barcelona, Spain; ^2^ Department of Nutrition, Food Science and Gastronomy, INSA-UB, Faculty of Pharmacy and Food Sciences, Universitat de Barcelona (UB), Barcelona, Spain; ^3^ Department of Biomedicine, Faculty of Medicine and Health Sciences, Universitat de Barcelona (UB), Barcelona, Spain

**Keywords:** exercise-induced muscle damage, fatigue, force-generating capacity, muscle recovery, repeated sprint ability

## Abstract

**Background:** Hamstring muscles are most affected by multiple sprint-based sports as a result of muscle strain during sprinting, leading to reduced performance and increased risk of injury. Therefore, the purpose of the study was to assess inter-individual variability in hamstrings recovery after a sport-specific repeated-sprint training (RST), through sprint-specific markers of muscle recovery and associated muscle damage biomarkers in women and men.

**Methods:** Healthy females (*n* = 14) and males (*n* = 15) underwent 10 repeated 40-m sprints with a 3-min rest pause between each repetition. Force-generating capacity (FGC) by the *90°*_*hip*_ *:20°*_*knee*_ *test* and range of motion *Jurdan test*, together with serum biomarkers [sarcomeric mitochondrial creatine kinase (sMtCK), oxidative stress, irisin] were tested at baseline and 24-, 48- and 72-h post-exercise through a repeated measures design. Participants were classified according to FGC loss into high responders (HR) and low responders (LR).

**Results:** 21 individuals (10 females, 11 males) were classified as HR (FGC loss >20% and recovery >48 h), while 8 individuals (4 females, 4 males) were classified as LR. HR individuals showed unrecovered maximal voluntary isometric contraction (MVIC) torque until 72 h post-training (*p* = 0.003, n_p_^2^ = 0.170), whereas only HR males showed decreased range of motion (*p* = 0.026, n_p_^2^ = 0.116). HR individuals also showed increased sMtCK (*p* = 0.016, n_p_^2^ = 0.128), oxidative stress (*p* = 0.038, n_p_^2^ = 0.106) and irisin (*p* = 0.019, n_p_^2^ = 0.123).

**Conclusion:** There is inter-individual variability in the muscular response to a sport-specific RST, identifiable by MVIC torque assessment. The findings support that the *90°*_*hip*_ *:20°*_*knee*_ *test* is a powerful indirect test to screen hamstrings recovery in both women and men, in a cost-effective way. However, the *Jurdan test* might not be able to monitor hamstrings recovery in sportswomen after RST. Decreases in muscle capacity are linked to damage to muscle sarcolemma and mitochondria until 72 h post-exercise. Overall, 72 h will not be adequate time to restore hamstrings structure and function after a sport-specific RST in both female and male responders.

## 1 Introduction

Repeated sprints, defined as periods of high-intensity running interspersed with lower intensity jogging and walking, are recurrent in multiple-sprint team sports, e.g., football, field hockey (Thompson et al., 1999). During sprinting, the muscle-tendon unit of the hamstring muscles reaches its maximum length in the late swing phase, while simultaneously undergoing an active lengthening eccentric contraction (Thelen et al., 2005). Thus, repeated high intensity eccentric contractions employed in repeated-sprint training (RST) result in loss of hamstring muscle function (Baumert et al., 2021). Muscle recovery is multifactorial in nature, as there are a wide range of intrinsic, e.g., training history, and extrinsic factors, e.g., seasonal moment, which influence the internal load suffered by athletes (Paul et al., 2015). The primary approach for assessing muscle recovery is muscle histology. However, this method is not used by professionals and practitioners due to its invasiveness and intrinsic errors of the approach (evaluation of the entire muscle by means of a microscopic muscle sample) (Clarkson and Hubal, 2002). Instead, there is a high correlation (*r* = 0.89) between the reduction in force-generating capacity (FGC) and the fraction of muscle fibers presenting ultrastructural alterations (Raastad et al., 2010). Consequently, FGC is the preferred indirect marker of muscle damage (MD) accounting for myofibrillar alterations, inflammation, and necrosis (Paulsen et al., 2012). Assessment of hamstring FGC has been commonly conducted using isometric posterior chain tests, such as the *90:90* or *90:30* supine test (Schache et al., 2011). However, as the largest decrease of isokinetic peak torque in the hamstring muscles appears in the most lengthened positions (10° of knee flexion), Matinlauri et al., 2018 recently designed a new, reliable, and sensitive test to monitor hamstring-related acute and residual fatigue after sprint-based exercises, the *90°*_*hip*_ *:20°*_*knee*_ *test*. The design of the test causes a lengthening of the hamstrings, and specifically, a greater biceps femoris hip extension than knee flexion moment arm, compared to the semitendinosus and semimembranosus muscles, in a similar pattern to the late swing phase of the running gait cycle (Thelen et al., 2005). Consequently, the hamstrings are biomechanically exposed during the test, thus facilitating hamstring-related acute and residual fatigue monitoring (Matinlauri et al., 2018).

Repeated high intensity eccentric contractions also result in changes of biochemical markers of MD and oxidative stress (OS), which indicate the status of myocyte structure (Brancaccio et al., 2010; Hyldahl and Hubal, 2014). Even there is no single gold-standard biomarker, a panel of biomarkers might be reported to simultaneously assessing different physiological processes and provide a more comprehensive understanding of muscle recovery. Mechanical-induced physical damage to membrane has been the underlying hypothesis explaining membrane permeability following eccentric actions. However, it has been found that increased intracellular calcium ion (Ca^2+^) over time as a consequence of stretch-activated Ca^2+^ channels plays a key role in the permeability of the sarcolemma (Allen et al., 2010). Considering a perspective applicable to the day-to-day sports practice, transient sarcoplasmic oxidation by-products and Ca^2+^ upregulation derived from damaging exercises results in a mild FGC loss as a consequence of failures in the excitation-contraction coupling process (Kano et al., 2012). Given that the mitochondria modulate Ca^2+^ overload through the mitochondrial calcium uniporter located in the inner mitochondrial membrane (Rossi et al., 2019), mild decreases in muscle function might be preserved by amino acids and/or micro-nutrients intake, e.g., Vitamin D, owning to the maintenance of the muscle mitochondria structural integrity and function (Drummond et al., 2009; Salles et al., 2022). However, persistent Ca^2+^ intracellular elevation activates the mitochondrial permeability transition pore (Bauer and Murphy, 2020) and upregulates calcium-dependent calpains (Belcastro et al., 1998). Calpains are predominantly localized in the Z-disk and I band regions, and have been shown to play a critical role in exercise-induced damage, as they are disruptive to the Z-disk and myofibrillar protein integrity (Hyatt and Powers, 2020). In particular, calcium-activated calpains impair contractile proteins, e.g., desmin, and/or excitation contraction coupling proteins, e.g., junctophilins (Hyatt and Powers, 2020). As a result, there is an uncontrolled release of Ca^2+^ and oxidation by-products to the sarcoplasm and bloodstream, finally resulting in rupture of the mitochondrial outer membrane and intermembrane proteins efflux (Görlach et al., 2015). However, the structural integrity of the muscle mitochondria can be monitored by measuring striated muscle mitochondrial-specific isoenzyme, sarcomeric mitochondrial creatine kinase (sMtCK) (Carmona et al., 2018). sMtCK is mainly localized in both the peripheral intermembrane space and the cristae space (Kottke et al., 1994; Schlattner et al., 2006). Considering the aforementioned localization of sMtCK, and that striated muscle mitochondria necrosis determined by histological evidence resulted in increased sMtCK in plasma, the efflux of sMtCK into the bloodstream reflects mitochondrial disruptions (Ishikawa et al., 1997). The afore-mentioned damage to cellular and mitochondrial integrity also leads to an upregulation of the signaling pathway for irisin cleavage (Liu et al., 2022). The myokine irisin plays a significant role in muscle remodeling by ameliorating protein degradation (Reza et al., 2017). Recently, irisin has also been shown to be involved in counteracting oxidative processes and proinflammatory cytokines (Mazur-Bialy and Pocheć, 2021). Considering the aforementioned irisin-mediated effects together with the fact that a recent study revealed that aerobic exercise-induced irisin partly mediates amelioration of oxidative stress and cell apoptosis in mice skeletal muscle (Ren et al., 2022), the myokine irisin is suggested to be increased during muscle recovery.

Finally, sarcomeres and mitochondria impairment caused by eccentric contractions during sprinting results in long-lasting decreases in FGC (Kano et al., 2012; Hyldahl and Hubal, 2014), which is a fundamental source for the loss of performance described in both sportswomen (Keane et al., 2015) and men (Howatson and Milak, 2009) after a RST. Accordingly, there is a need for practitioners to monitor FGC and range of motion (ROM) using simple, time and cost-effective sprint-specific muscle function tests associated with biochemical markers using an integrative-based approach. To our knowledge, the use of the *90°*_*hip*_ *:20°*_*knee*_ *test* to monitor hamstrings FGC during the recovery period after RST is described for the first time. Therefore, the purpose of the research was to assess inter-individual variability in hamstrings recovery after a sport-specific RST, through sprint-specific markers of muscle recovery and associated muscle damage biomarkers in women and men.

## 2 Materials and methods

### 2.1 Participants

Fifteen healthy males [age = 22.5 ± 3.2 years, mass = 74.2 ± 7.4 kg, height = 175.9 ± 6.8 cm (mean ± SD)] and fourteen females [age = 22.2 ± 3.1 years, mass = 60.3 ± 7.1 kg, height = 165.2 ± 4.8 cm (mean ± SD)] voluntarily attended the study during 2021 and 2022 spring. Participants were required to be physically active according to the World Health Organization (at least 150 min of moderate-intensity exercise per week), but not enrolled in a regular specialized training program. Exclusion criteria were non-healthy participants and lower limb muscle injuries within 6 months prior to the experiment. Participants were strongly encouraged to (i) avoid any type of physical exercise during the 3 days prior to the experiment and during the 72-h follow-up, and (ii) to maintain their daily diet and lifestyle. All participants were previously informed and gave their written consent that they received complete and specific information about the purpose of the study, experimental tests, and associated risks. The experiment was conducted in accordance with the code of ethics of the World Medical Association (Declaration of Helsinki) and was approved by the Ethics Committee for Clinical Research of the Catalan Sports Council (Generalitat de Catalunya) (037/CEICGC/2021).

### 2.2 Experimental design

**FIGURE 1:**
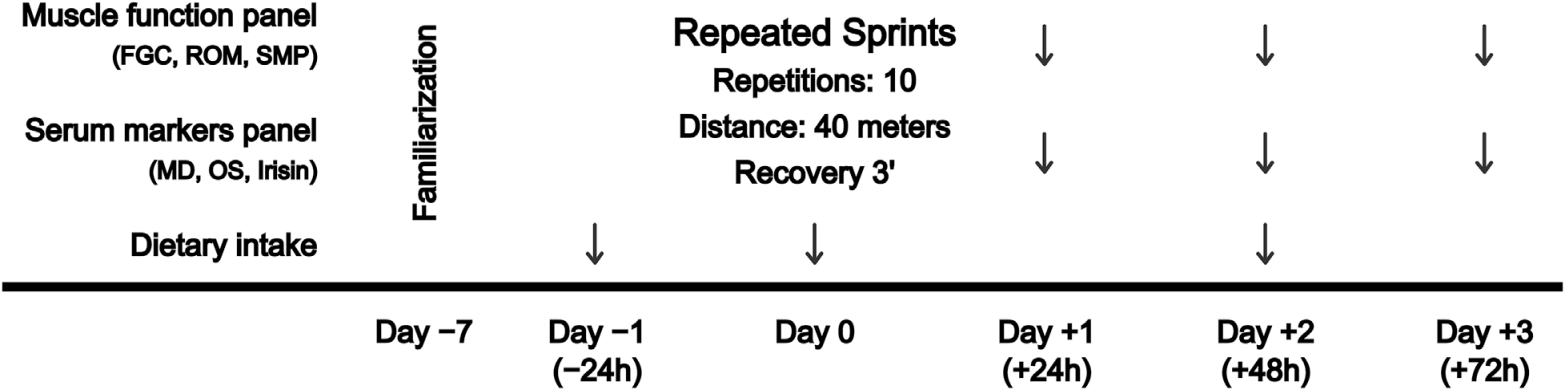
Schematic overview showing the experimental design. FGC, force-generating capacity; MD, muscle damage; OS, oxidative stress; ROM, range of motion; SMP, sprint mechanical properties.

A repeated-measures design was used to examine the extent and time course of serum biomarkers and muscle function before (pre) and at 24 (+24 h), 48 (+48 h) and 72 h (+72 h) after a RST, consisting of 10 repeated 40-m sprints, with a 3-min rest between each repetition. Depending on the playing position, sprint distance in football matches ranges from 190 to 420 m (Altmann et al., 2021), in field hockey matches from 418 to 513 m (Sunderland and Edwards, 2017), and in rugby matches from 65 to 392 m (Jones et al., 2015). In addition, similar RST protocols (3 sets x 6 repetitions x 20 m = 360 m, rest: 1.5 min between repetitions and 4 min between sets) have shown decreases in hamstrings electrical activity and knee flexors torque (Timmins et al., 2014). Accordingly, the total training distance (400 m) was determined based on the aforementioned sprint demands of outfield team sports, and the distance of each sprint (40 m) was selected as it allows reaching the maximum sprint velocity, which is the main injury mechanism of hamstrings strains (Danielsson et al., 2020). Long rest periods between repetitions were selected to reduce intra-session fatigue and maintain the maximum intensity in each 40-m repetition. The designed protocol is further supported by the findings of a recent systematic review and meta-analysis on the demands of RST in team sports (Thurlow et al., 2023), which include that (i) a distance of 40 m (or 6-s sprints), commonly used in team sports, allows the maximum velocity to be reached; and (ii) longer rest intervals are recommended in order to maintain the quality of the repetitions when the total volume is greater. Then, blood samples were collected to determine changes in serum biomarkers, and muscle function was assessed by posterior chain FGC, lower limbs ROM and sprint performance and mechanical properties test. Participants completed the familiarization 1 week before the start of the experimental procedure. All functional tests were repeated by following the instructions of the researcher until successful completion. A schematic overview of the experimental design is shown in Figure 1.

To further explain the findings given the inter-individual variations in response to exercise-induced MD, participants were categorized according to the percentage of reduction of the FGC during the 3-day follow up (Paulsen et al., 2012). Then, participants were grouped into “high-responders” (HR), participants who showed notable decreases in FGC of 20%–50% of baseline values and recovery after more than 48 h, and “low-responders” (LR), participants who showed FGC decreases of <20% and complete recovery within 48 h (Paulsen et al., 2012).

### 2.3 Force-generating capacity (FGC)

FGC of hamstring muscles was measured unilaterally as the maximal voluntary isometric contraction (MVIC) torque by the *90°*_*hip*_ *:20°*_*knee*_ *test* (Matinlauri et al., 2018), using a 160 Hz sampling frequency strain gauge (Chronojump, Barcelona, Spain). Participants were verbally encouraged to exert their maximum effort and completed three trials (3–5 s MVIC) of the test with each lower limb, with a 30-s rest between trials. The maximum MVIC torque was registered as the average force in a 1-s window once a force plateau had been established (Tesch et al., 2004) and the residual force resulting from the mass of the tested leg at rest was then subtracted. The best of the three trials from each limb was recorded for further analyses. Additional trials were conducted if any countermovement or joint balancing was noted.

### 2.4 Range of motion (ROM)

The *Jurdan test* was used to evaluate de interaction between hip flexor and hamstring flexibility, measured as the differential angle between the thigh and contralateral shin (Lahti et al., 2020). The test was photographically registered by a SONY DSC-RX100M5A placed on a tripod located at the height of the table and 3 m from the center of the hip of the participant. 2D shin and thigh angles were measured, relative to the horizontal, as the best of two trials on each lateral approach using the sports kinematics analysis software *Kinovea* v0.9.3. Then, resulting angles were used to compute a composite angle defined as the difference between the actively lengthened leg shin angle and the opposite leg passive thigh angle.

### 2.5 Sprint performance and mechanical properties

Instantaneous velocity over 40 m-sprints was measured by a friction encoder *RaceAnalyzer* (Chronojump, Barcelona, Spain), with a distance accuracy of 3 cm and temporal accuracy of 4 microseconds. The device was located on a 1-m-high tripod placed behind the participants. Then, participants were positioned into a belt attached to the friction encoder and were strongly verbally encouraged during the test to ensure maximal effort. Data were *post hoc* analyzed using Chronojump software v2.1.2-2 (Chronojump, Barcelona, Spain) to compute sprint F-V spectra using the validated method described in detail elsewhere (Samozino et al., 2016). Individual linear F-V relationships were then extrapolated to calculate theoretical maximal running velocity (V0), relative (to body mass) theoretical maximal horizontal force production (F0.rel), peak acceleration (A.max), relative peak power (P.max.rel), time to peak power (Time.P.max) and rate of force development normalized by peak force (K.fitted).

### 2.6 Blood sampling and processing

An 8-mL blood sample was drawn from an antecubital vein. Samples were allowed to clot for 30 min at room temperature in an SST II Advance tube (Becton Dickinson Vacutainer Systems, UK) and were then centrifuged at 4,000 g for 8 min at 4°C. After separation of the serum, aliquots were stored at −80°C until analysis. Measurement of creatine kinase (CK) activity was conducted on an Advia 2400 automated device (Siemens™ Medical Solutions Diagnostics, Tarrytown, NY, United States) and serum sMtCK was measured by ELISA kit #SEC386Hu (Cloud Clone Corp., Houston, TX, United States) according to manufacturer’s instructions (intra-assay coefficient of variability (CV) < 10%; inter-assay CV < 12%; minimum detectable value of 1.56 ng mL^-1^). The Erel approach (Erel, 2005) was used to quantify the oxidizing material in the samples, based on the oxidation of ferrous iron to ferric iron with an intra-assay CV of 2.3%, inter-assay CV of 3.5% and a minimum detectable value of 2.5 μmol H_2_O_2_ Equiv.·mL^-1^ (Elabscience #E-BC-K802-M, Wuhan, China). Similarly, the Erel 2,2′-azino-di-3-ethylbenzthiazoline sulfonate (ABTS)+ approach (Erel, 2004) with an intra-assay CV of 4.6%, inter-assay CV of 7.0% and a minimum detectable value of 0.23 mmol Trolox Equiv.·mL^−1^ (Elabscience #E-BC-K801-M, Wuhan, China) was conducted to quantify total antioxidant status (TAS). Then, OS index (OSI) was calculated as the oxidizing material (TOS) to TAS ratio. In order to control the OSI results, serum malondialdehyde (MDA) was measured by kit #E-EL-0060 (Elabscience, Wuhan, China) with an intra-assay CV < 10%, inter-assay CV < 10% and a minimum detectable value of 18.75 ng mL^−1^. Circulating irisin was quantified using the previously validated kit against mass spectrometry (Ruan et al., 2019) #EK-067-29 (Phoenix Pharmaceuticals Inc, Burlingame, CA, United States), according to manufacturer’s instructions (intra-assay CV < 10%; inter-assay CV < 15%; minimum detectable value of 1.29 ng mL^−1^). 25-hydroxyvitamin D (25OHD) concentration was measured by chemiluminescence immunoassay procedures conducted on a *Advia Centaur Vitamin D Total Assay* (Siemens™ Healthcare Diagnostics, Tarrytown, NY, United States). The method is certified by the Centers for Disease Control and Prevention and adhered to de Vitamin D Standardization-Certification Program (Binkley and Sempos, 2014).

### 2.7 Dietary intake record

Participants were instructed to record their diet for 3 days (−24 h, 0 h and +48 h) and were given detailed verbal and written instructions describing procedures for recording the daily diet, together with a list of commonly used household measurements (e.g., dessert spoon, glass, cup) and measurements quantities (e.g., grams, milliliters). The participants were strongly instructed to maintain their usual diet, and to be accurate in recording the amounts and types of food and drinks intake. The diet of each participant was calculated as the 3-day average by using the dietary analysis software *PCN Pro v1.* (Universitat de Barcelona, Barcelona, Spain).

### 2.8 Statistical analysis

First, sample size was estimated using G*Power software (version 3.1) based on MVIC change (*90°*_*hip*_ *:20°*_*knee*_ *test*) of the study by Matinlauri et al. (2018). A total of 25 participants were calculated to be included given an effect size of 0.68, a power of 90% and a 5% alpha error. Asymmetrically distributed variables were log-transformed prior to analysis. A three-way repeated measures ANOVA [Time (baseline, 24h, 48h, and 72 h) x Sex (male, female) x Group (HR, LR)] was used to identify the effect of time on MVIC torque, ROM, sprint performance and mechanical properties and serum biomarkers, together with interactions between sex and groups. Post-hoc Bonferroni-corrected paired *t*-test were conducted to identify significant differences from baseline values. Effect sizes (ES) were calculated using Cohen’s d to assess the difference between means when statistically significant differences were found. Thresholds for standardized differences statistics were (<0.20), trivial; (0.20 < 0.59), small; (0.60 < 1.19), moderate; (1.20 < 1.99), large; and (>2.0), very large (Hopkins et al., 2009). Associations between variables of interest were analyzed using Spearman’s Rho correlation coefficient, considering the following thresholds (<0.30), small; (0.30 < 0.51), moderate; (0.51 < 0.70), large; (0.71 < 0.90), very large; and (>0.90), extremely large (Hopkins et al., 2009). The intraclass correlation coefficient (ICC) was used to assess the reliability of a single data collector that examines the degree of concordance of scores between repetitions or sessions. The ICC was calculated for average measures using a 2-way random effect model of absolute agreement. Thresholds for ICC were (0.00 < 0.50), poor; (0.51 < 0.75), moderate; (0.46 < 0.90), good; and (0.91 < 1.00), excellent (Koo and Li, 2016). Independent samples *t*-test were used to assess between-groups differences in dietary intakes. Data are presented as mean ± standard deviation (SD) and the level of significance was set at *p* < 0.05. Statistical analysis was performed with *SPSS version 27.0.1.0* (SPSS Statistics, IBM Corp., Armonk, NY, United States).

## 3 Results

According to the percentage of reduction in FGC (Paulsen et al., 2012), 21 individuals (10 females, 11 males) were classified under HR conditions, as they presented notable decreases in FGC of 20%–50% of baseline values and recovery after more than 48 h. Otherwise, 8 individuals (4 females, 4 males) showed FGC decreases of <20% and complete recovery within 48 h and were therefore classified as LR.

### 3.1 Muscle function

#### 3.1.1 MVIC torque

**FIGURE 2:**
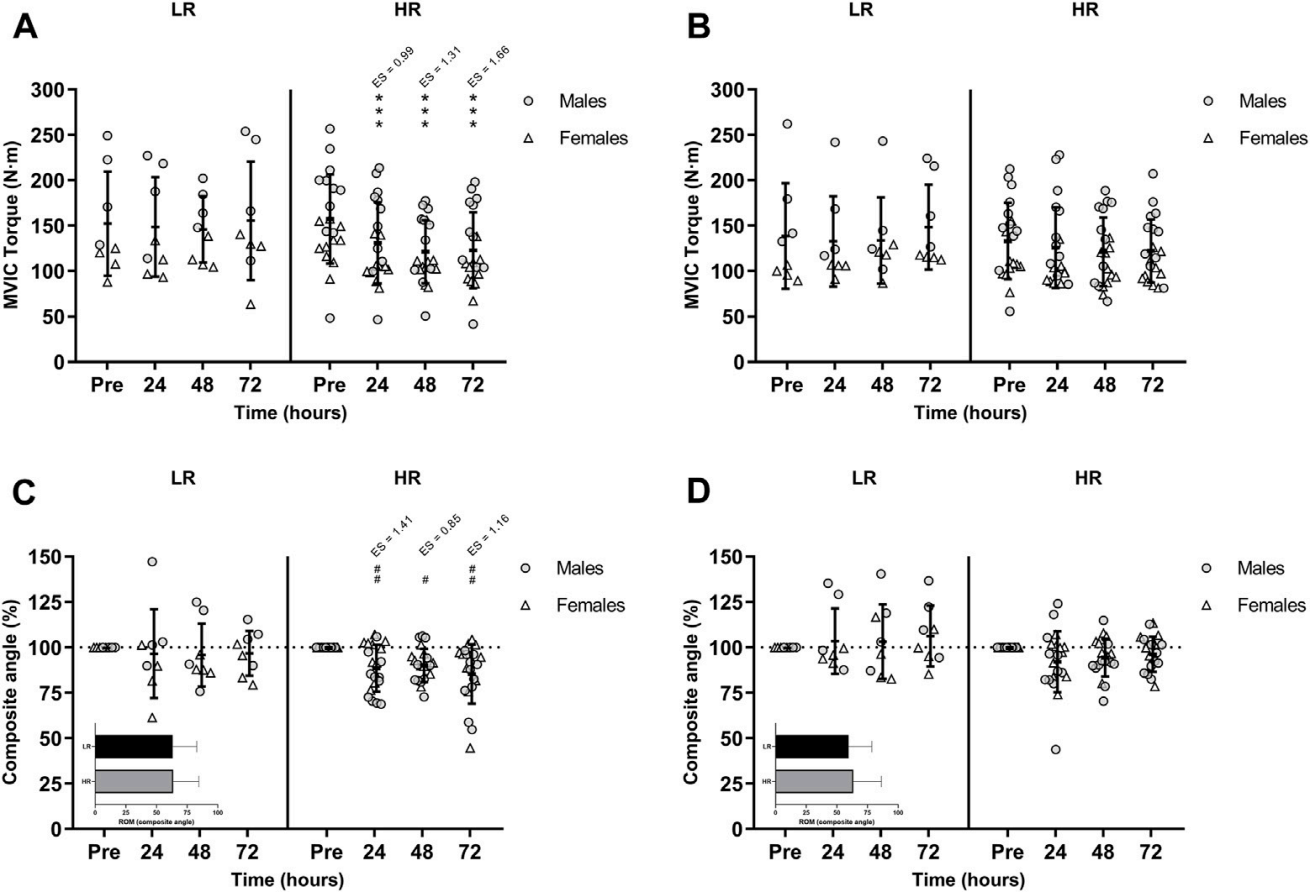
Maximal voluntary isometric contraction (MVIC) torque of the lower-limb showing **(A)** the larger and **(B)** the smaller reduction of MVIC torque during recovery period; and percentages of the range of motion (ROM) composite angle of **(C)** the most damaged and **(D)** the least damage lower-limb during recovery period. Data are expressed as mean ± SD. Baseline values of ROM composite angle are shown in the inset graphs. (*), (**), and (***) indicate a significant difference from the baseline value at *p* < 0.05, *p* < 0.01, and *p* < 0.001, respectively. (#), (##), and (###) indicates a significant difference from the baseline value in males. ES, effect size; HR, high responders; LR, low responders; N·m, newtons-meter.

The ICC was excellent for the dominant leg with a value of 0.973 (95% IC 0.941–0.988) and non-dominant leg with a value of 0.969 (95% IC 0.937–0.986). As illustrated in Figure 2A and Figure 2B, hamstring MVIC torque was decreased and unrecovered in HR individuals during the 72-h recovery period. There was no sex-related time course difference in MVIC torque.

#### 3.1.2 Range of motion

There was a significant time-group-sex interaction in the composite ROM following RST [F(3,75) = 3.277, *p* = 0.026, n^2^_p_ = 0.116]. Post-hoc Bonferroni-corrected paired *t*-test showed unchanged ROM in LR group, while ROM was only decreased in males HR group during the 72-h recovery period (Figure 2C and Figure 2D).

#### 3.1.3 Sprint performance and mechanical properties

The RST increased split time to 10 m and total time to 40 m during recovery period. Also, Both the LR and HR individuals showed decreased F0.rel, A.max, P.max.rel, K.fitted and increased Time.P.max during the post-RST recovery period. V0 was only decreased in HR individuals. There were no group and sex-related time course differences (Table 1).

**TABLE 1:** Sprint performance and mechanical properties during post-exercise recovery period.

Parameter	Group	Pre	+24 h	+48 h	+72 h
**Time to 10** **m (s)**	LR	2.185 ± 0.085	2.297 ± 0.184	2.415 ± 0.209^***^	2.407 ± 0.222^**^
	HR	2.171 ± 0.114	2.316 ± 0.173^***^	2.288 ± 0.126^**^	2.289 ± 0.149^**^
	**ALL**	**2.175 ± 0.106**	**2.311 ± 0.173** ^***^	**2.323 ± 0.160** ^***^	**2.321 ± 0.176** ^***^
**Time to 40** **m (s)**	LR	6.178 ± 0.387	6.242 ± 0.473	6.465 ± 0.472^**^	6.530 ± 0.471^**^
	HR	6.217 ± 0.569	6.416 ± 0.548^**^	6.402 ± 0.513^***^	6.417 ± 0.549^**^
	**ALL**	**6.207 ± 0.519**	**6.368 ± 0.526** ^*^	**6.420 ± 0.494** ^***^	**6.448 ± 0.523** ^***^
**V0 (m·s** ^**-1**^ **)**	LR	8.341 ± 0.801	8.314 ± 0.758	8.216 ± 1.029	8.034 ± 0.693
	HR	8.256 ± 1.117	7.992 ± 0.943^**^	7.943 ± 0.988^**^	7.926 ± 0.984^**^
	**ALL**	**8.280 ± 1.026**	**8.081 ± 0.895**	**8.018 ± 0.989** ^*^	**7.956 ± 0.903** ^**^
**F0.rel (N·Kg** ^**-1**^ **)**	LR	7.608 ± 0.582	6.732 ± 0.995	6.268 ± 1.118^***^	6.313 ± 1.660^**^
	HR	7.790 ± 0.740	6.717 ± 0.922^***^	7.042 ± 0.845^***^	6.953 ± 1.004^**^
	**ALL**	**7.740 ± 0.695**	**6.721 ± 0.924** ^***^	**6.829 ± 0.973** ^***^	**6.776 ± 1.222** ^***^
**A.max (m·s** ^**-2**^ **)**	LR	7.677 ± 0.580	6.806 ± 1.002	6.339 ± 1.118^***^	6.382 ± 1.663^**^
	HR	7.859 ± 0.745	6.787 ± 0.929^***^	7.112 ± 0.849^***^	7.022 ± 1.008^**^
	**ALL**	**7.809 ± 0.698**	**6.792 ± 0.931** ^***^	**6.898 ± 0.975** ^***^	**6.845 ± 1.226** ^***^
**P.max.rel (W·Kg** ^**-1**^ **)**	LR	15.097 ± 1.668	13.972 ± 2.919	12.735 ± 2.577^***^	12.587 ± 3.568^**^
	HR	15.287 ± 2.663	13.407 ± 2.869^***^	13.897 ± 2.501^**^	13.723 ± 2.769^**^
	**ALL**	**15.235 ± 2.402**	**13.563 ± 2.842** ^**^	**13.577 ± 2.531** ^***^	**13.410 ± 2.988** ^***^
**Time.P.max (s)**	LR	0.721 ± 0.112	0.863 ± 0.128^*^	0.931 ± 0.238^***^	0.926 ± 0.245^***^
	HR	0.698 ± 0.100	0.832 ± 0.140^***^	0.791 ± 0.139^**^	0.800 ± 0.132^**^
	**ALL**	**0.704 ± 0.102**	**0.840 ± 0.135** ^***^	**0.830 ± 0.179** ^***^	**0.835 ± 0.175** ^***^
**K.fitted (s** ^**-1**^ **)**	LR	1.011 ± 0.144	0.850 ± 0.112^**^	0.812 ± 0.165^***^	0.829 ± 0.217^**^
	HR	1.047 ± 0.155	0.885 ± 0.123^***^	0.938 ± 0.162^***^	0.927 ± 0.172^**^
	**ALL**	**1.037 ± 0.150**	**0.876 ± 0.120** ^***^	**0.903 ± 0.170** ^***^	**0.900 ± 0.187** ^***^

Data are expressed as mean ± SD. ^✝^Significantly different between groups at baseline. (*), (**), and (***) indicate a significant difference from the baseline value at *p < 0.05*, *p < 0.01*, and *p < 0.001*, respectively. Bold values represent the data for the complete sample, considering high responders and low responders together. A.max, peak acceleration; F0.rel, relative theoretical maximal horizontal force production; HR, high responders; K.fitted, rate of force development normalized by peak force; Kg, kilograms; LR, low responders; m, meters; N, newtons; P.max.rel, relative peak power; s, seconds; Time.P.max, time to peak power; V0, theoretical maximal running velocity; W, watts.

### 3.2 Biochemical measures

**FIGURE 3:**
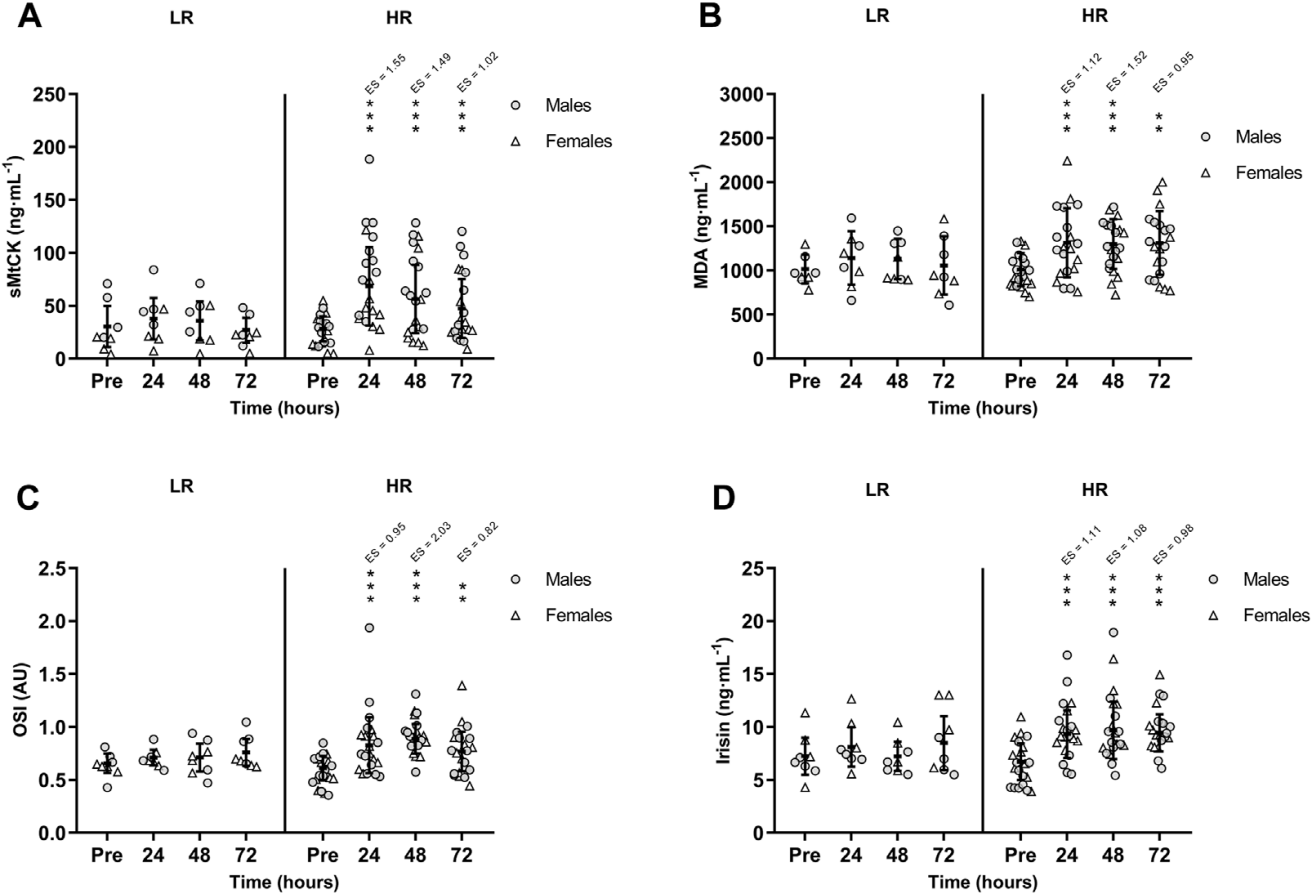
Mean (±SD) values of **(A)** Sarcomeric Mitochondrial Creatine Kinase (sMtCK); **(B)** Malondialdehyde (MDA); **(C)** Oxidative Stress Index (OSI); and **(D)** Irisin. (*), (**), and (***) indicate a significant difference from the baseline value at *p* < 0.05, *p* < 0.01, and *p* < 0.001, respectively. AU, arbitrary units; ES, effect size; HR, high responders; LR, low responders.

CK activity remained unchanged in the LR group, while CK was peaked in HR group at +24 h (*p* < 0.001; ES = 1.77) and remained increased at +48 h (*p* < 0.001; ES = 1.50) and at +72 h (*p* < 0.001; ES = 1.02). Also, there was a significant time-group interaction in serum sMtCK values [F(3,75) = 3.665, *p* = 0.016, n^2^_p_ = 0.128] following RST (Figure 3A). MDA and OSI were only increased in HR individuals and remained elevated during recovery period (Figures 3B, C). Similarly, HR participants showed increased TOS at +24 h (*p* = 0.002; ES = 0.91), +48 h (*p* < 0.001; ES = 1.83) and +72 h (*p* = 0.002; ES = 0.83). However, TAS recovered basal values at +72 h. Finally, there was a significant time-group interaction in circulating irisin values following RST [F(3,75) = 3.505, *p* = 0.019, n^2^_p_ = 0.123]. Post-hoc Bonferroni-corrected paired *t*-test showed unchanged irisin in LR group, while irisin was increased in HR group at +24 h, peaked at +48 h, and remained increased at +72 h (Figure 3D). There were no between-sex differences in the interaction of time and group for CK, sMtCK MDA, OSI, TOS, TAS and irisin.

Table 2 shows the correlation matrix between changes in serum biomarkers and daily dietary intakes. CK and sMtCK increases were negatively and moderately correlated with daily total protein intake (*p* = 0.028 and *p* = 0.007; respectively). In addition, there was a strong correlation between the extent of damage to muscle mitochondria, oxidative stress and irisin.

**TABLE 2:** Spearman’s rank correlation coefficient matrix of peak fold-changes in serum biomarkers and daily dietary intakes (*n* = 29).

	CK	sMtCK	MDA	OSI	TOS	TAS	Irisin	TE	TC	TPI	API	PPI
**CK**	—											
**sMtCK**	0.663*	—										
**MDA**	0.197	0.201	—									
**OSI**	0.383*	0.595*	0.238	—								
**TOS**	0.375*	0.382*	0.230	0.796*	—							
**TAS**	−0.277	−0.442*	−0.185	−0.626*	−0.159	—						
**Irisin**	0.262	0.554*	0.123	0.618*	0.390*	−0.372*	—					
**TE**	0.074	−0,130	−0.115	−0.185	−0.163	0.134	−0.174	—				
**TC**	0.099	0,011	−0.015	−0.260	−0.171	0.165	−0.021	0,753*	—			
**TPI**	−0.423*	−0.511*	−0.150	−0.261	−0.213	0.234	−0.503*	**0.673**	**0.404**	—		
**API**	−0.350	−0.460*	−0.202	−0.146	−0.200	0.150	−0.360	**0.667**	0.277	0.940*	—	
**PPI**	−0.048	−0.033	0.238	−0.068	−0.013	−0.255	−0.348	0.200	0.405*	0.267	0.029	—

(*) indicates a significant correlation at *p* < 0.05. API, animal-based protein intake; CK, creatine kinase; MDA, malondialdehyde; OSI, oxidative stress index; PPI, plant-based protein intake; sMtCK, sarcomeric mitochondrial creatine kinase; TAS, total antioxidant status; TC, total carbohydrates intake; TE, total energy intake; TOS, total oxidant status; TPI, total protein intake.

The significant correlations are in bold type for a clearer visualization of the data set.

### 3.3 Dietary intake

There were no differences between HR and LR individuals in energy intake, neither in macronutrient nor micronutrient intake (Table 3). Also, there were no statistically significant differences between HR group (29.4 ± 9.0 ng mL^−1^) and LR group (26.7 ± 9.0 ng mL^−1^) in serum 25OHD status during the experimental protocol (MD = 2.77 ng mL^−1^; t = 0.737; *p* = 0.468).

**TABLE 3:** Descriptive summary of macro- and micronutrient intakes.

	HR (*n* = 21)	LR (*n* = 8)
**Total energy** (kcal·day^-1^)	2163.6 ± 538.6	2281.8 ± 594.1
**Total Protein Intake** (g·Kg^-1^·day^-1^)	1.67 ± 0.30	1.91 ± 0.66
**Animal-based Protein Intake** (g·Kg^-1^·day^-1^)	1.22 ± 0.27	1.39 ± 0.53
**Plant-based Protein Intake** (g·Kg^-1^·day^-1^)	0.42 ± 0.10	0.41 ± 0.07
**Total carbohydrates** (g·day^-1^)	210.0 ± 56.3	215.3 ± 37.7
**Total lipids** (g·day^-1^)	96.8 ± 34.8	94.5 ± 30.7
**Water** (g·day^-1^)	2388.6 ± 773.4	2436.8 ± 722.7
**Vitamin C** (μg·day^-1^)	117.46 ± 60.75	116.02 ± 55.58
**Vitamin D** (μg·day^-1^)	4.31 ± 4.74	4.06 ± 3.85
**Vitamin E** (mg·day^-1^)	8.87 ± 3.00	8.96 ± 3.99

Data are expressed as mean ± SD. *Significantly different between groups, independent samples *t*-test, *p* < 0.05. HR, high responders; LR, low responders.

## 4 Discussion

Novel sprint and hamstring-specific markers of muscle recovery were monitored during 72 h after a sport-specific RST in women and men. The findings of the research highlight that (i) there is heterogeneity in the muscular response to an RST, identifiable by MVIC torque assessment; (ii) the *90°*_*hip*_ *:*20°_knee_ test for males and females, and the *Jurdan test* for males, could be powerful indirect tests to screen hamstring muscle recovery after a sport-specific RST; (iii) there is a release of sMtCK into the bloodstream during recovery period, potentially related to mitochondrial dysfunction, or mitochondrial membrane permeability/rupture; and (iv) oxidative stress and circulating irisin are upregulated during the 72-h recovery period in both HR females and males.

### 4.1 Hamstring muscle capacity is impaired during recovery within high responders

It has been widely reported that high-velocity eccentric contractions employed in RST leads to increased muscle-specific proteins leakage into the bloodstream, muscle soreness, as well as reduced performance during 48–72 h in both collegiate team-sportswomen (Keane et al., 2015) and men (Howatson and Milak, 2009). However, to further explain the results given the within-human heterogeneity in response to exercise training and adaptation, individuals were categorized according to the percentage of FGC loss (Paulsen et al., 2012). HR participants (*n* = 21) showed an average hamstring FGC decrease of 21.7% at +72 h, while the LR individuals (*n* = 8) showed non-significant changes in hamstring FGC. Given the well-established correlation between ultrastructurally damaged myocytes and FGC (Raastad et al., 2010), sprint-specific muscle function tests were examined to determine muscle recovery. Previous results after RST showed decreases in quadriceps MVIC until +48 h in men (Howatson and Milak, 2009), while there were no decreases in women (Keane et al., 2015). However, the *90°*_*hip*_ *:20°*_*knee*_ *test* reported decreases in hamstring MVIC torque at +24 h, +48 h and +72 h post-RST in HR individuals, whereas LR participants showed no significant differences during the recovery period. Loss of performance in the *90°*_*hip*_ *:20°*_*knee*_ test represents a reduced FGC in the late swing phase derived from disrupted sarcomeres (Matinlauri et al., 2018), which is the phase in which most hamstring injuries occur as it is biomechanically exposed (Chumanov et al., 2012). Then, our data suggest that the *90°*_*hip*_ *:20°*_*knee*_ *test* can be used to monitor hamstring muscle recovery in both women and men.

On the other hand, the *Jurdan test* showed decreases in the ROM of HR males until 72 h post-RST. LR individuals, as well as HR females, showed no differences in ROM. Anatomical differences between females and males, e.g., muscle mass size, joint geometry, and physiological differences, e.g., greater hamstring extensibility, lower passive stiffness, better stretch tolerance, result in women usually exhibiting a greater ROM (Marshall and Siegler, 2014). Also, considering that the *Jurdan test* is an indicator of increased passive tension derived from damage to the sarcolemma of the iliopsoas and/or hamstring muscles (Lahti et al., 2020), and the aforementioned between-sex differences, females might be less likely to experience ROM loss. Therefore, although HR females showed impaired muscle structure and function, the *Jurdan test* may not show such impairment after RST. Finally, considering the characteristics of the damaging model, sprint testing is the most specific test to determine performance recovery. There was a degradation of sprint times and mechanical properties in both HR and LR participants. Only V0 was decreased in HR compared to LR individuals during the post-RST recovery period, accounting for a horizontal FGC loss at high velocities. This finding may be attributed to the fact that the hamstring muscles are the key mediators of the forward orientation of the ground reaction force during the sprint acceleration phase (Morin et al., 2012; Morin et al., 2015). Overall, HR individuals, as determined by MVIC torque loss, also showed decreased ROM and horizontal FGC during the sprint acceleration phase until +72 h, with no differences between sex.

### 4.2 Is loss of hamstring muscle capacity linked to serum biomarkers?

Blood biomarkers further support the analysis of exercise-induced damage and muscle recovery (Bessa et al., 2016), since the structural status of myocytes can be assessed by changes in biochemical markers (Brancaccio et al., 2010). Peak CK activity observed in this study (723 ± 526 IU L^−1^) is consistent with other similar RST studies (776 ± 312 IU·L^−1^) (Howatson and Milak, 2009), indicating a permeability and/or breakage of the sarcolemma (Brancaccio et al., 2010; Hyldahl and Hubal, 2014). The lipid peroxidation marker MDA was also moderately increased during the recovery period (ES = 0.95), further supporting oxidative damage to the sarcolemma. Sarcolemma impairment-derived sarcoplasmic Ca^2+^ concentration is modulated by mitochondrial Ca^2+^ uptake (Rossi et al., 2019). Excessive Ca^2+^ accumulation in the mitochondrial matrix leads to a rupture of the mitochondrial outer membrane and intermembrane content efflux, i.e., sMtCK, (Görlach et al., 2015). In this way, the muscle mitochondria were moderately damaged in HR individuals until +72 h (ES = 1.02), while no changes were observed in LR individuals. Muscle mitochondria play a critical role during skeletal muscle repair, as it requires a substantial energy supply, and mitochondria are the primary source of energy production. In this sense, satellite cell signaling, which is a key factor in muscle repair, can be regulated by the mitochondria (Alway et al., 2023a). Mitochondria have also been shown to be essential in the processes of myoblast differentiation and migration into damaged fibers since mitochondrial dynamics disorders are an impediment to correct myogenic differentiation (Kim et al., 2013). Further, transplantation of healthy mitochondria into damaged skeletal muscle of murine models improved myocyte repair and recovery of muscle function (Alway et al., 2023b). Thus, given that skeletal muscle repair is limited by mitochondrial dysfunction, RST-derived damage to muscle mitochondria is a key aspect to monitor during hamstring muscle recovery through sMtCK assessment. There is also an outflow of reactive oxygen species (ROS) to the myocyte and bloodstream (Görlach et al., 2015) given that both the sarcolemma and mitochondria of the HR individuals were affected by the sport-specific RST. Since OS is a dynamic process involving large number of pro-oxidant components, e.g., total hydroperoxides, MDA, and anti-oxidant components, e.g., superoxide dismutase, glutathione peroxidase, integrative-based quantification methods such as OSI should be used to fully understand the complex interaction between exercise and OS (Thirupathi et al., 2021). Separate measurement of different oxidant and anti-oxidant molecules is complex, time-consuming, and the oxidant or antioxidant effects are additive. However, OSI considers the measurement of both oxidant and antioxidant activity produced by the action of multiple components measured as one (Sánchez-Rodríguez and Mendoza-Núñez, 2019). RST led to a moderate OS state (ES = 0.82) until +72 h post-RST. There was also a notable correlation between OS and damage to muscle mitochondria, explained by the significant implication of mitochondria in the efflux of ROS into the bloodstream after damaging exercises (Görlach et al., 2015).

Repeated muscle contractions during the RST and the afore-mentioned mechanical and oxidative damage to cellular and mitochondrial integrity leads to an upregulation of the AMP-activated protein kinase (AMPK) - peroxisome proliferator-activated receptor γ co-activator 1α (PGC-1α) - fibronectin domain-containing 5 protein (FNDC5) signaling axis pathway, and subsequent irisin cleavage (Boström et al., 2012; Liu et al., 2022). The myokine irisin plays an important role in muscle remodeling by stimulating satellite cell activation and ameliorating protein degradation (Reza et al., 2017). Also, irisin has been shown to be involved in counteracting oxidative processes related to free radical production (Mazur-Bialy, 2017). Irisin plays a role in inhibiting the Toll-like receptor 4/Myeloid differentiation factor 88 downstream signaling pathway, resulting in reduced activation of nuclear factor kappalight-chain-enhancer of activated B cells (NF-κB) (Mazur-Bialy et al., 2017). As a result of depressed NF-κB, irisin downregulates the expression of tumor necrosis factor-alpha, interleukin (IL)-6 and IL-1β proinflammatory cytokines (Mazur-Bialy et al., 2017) and promotes the polarization of pro-inflammatory M1 into anti-inflammatory M2 macrophages (Dong et al., 2016). The above-mentioned irisin-mediated effects together with the irisin-induced inhibition of ROS-NLRP3 inflammasome pathway led to an attenuated oxido-inflammatory response (Deng et al., 2018). Furthermore, muscle-damaging exercise leads to the release of irisin into the bloodstream in humans immediately after a single bout of exercise (Huh et al., 2015). Accordingly, the findings of the present study revealed a moderate increase in circulating irisin over 50.0% (ES = 0.98) during the 72-h recovery period. Considering that irisin reduces the activity of ROS-producing processes (Mazur-Bialy, 2017), downregulating ROS-NLRP3 inflammasome signaling (Deng et al., 2018), and that aerobic exercise-induced irisin partly mediates amelioration of OS and cell apoptosis in mice skeletal muscle (Ren et al., 2022), we suggest that increased circulating irisin during recovery period is a counteracting mechanism to reduce exercise-induced MD and OS. Further, the findings showed statistically large and positive correlations between irisin, and both OSI and sMtCK. Hence, exercise-induced MD and oxidative processes might be a trigger for irisin synthesis to counteract muscle cell damage.

### 4.3 Inter-individual heterogeneity in hamstring muscle recovery

The LR individuals showed no changes in blood markers of MD/OS, nor in muscle function during recovery period. This between-group heterogeneity might be related to endogenous factors, e.g., age, training status, and/or exogenous factors, e.g., volume, intensity, and type of exercise (Paulsen et al., 2012). In this study, all the individuals were young, healthy, physically active, and with no previous experience in eccentric exercising, in addition to undergoing the same standardized training. Therefore, it is assumed that the response heterogeneity is not attributable to these factors. However, inter-individual variations in response to exercise are in most cases due to genetics and/or dietary intake (Mann et al., 2014). Physical exercise together with protein intake modulates muscle protein synthesis, as a result of increased bioavailability of exogenous essential amino acids (Drummond et al., 2009). As a result, upregulated muscle protein synthesis during the recovery period after acute endurance exercise promotes muscle adaptation and structural protein repair, facilitating FGC recovery (Howarth et al., 2009). Other recent applied studies have shown that increasing protein intake enhances functional recovery after sprinting training (Kritikos et al., 2021). In this way, our findings showed that both total protein intake and animal-based protein intake were moderately and inversely correlated with MD markers. Thus, we suggest that increased daily protein intake during the peri-exercise days might protect LR individuals. Finally, vitamin D intake and/or 25OHD serum levels also play a protective role on mitochondrial membranes (Salles et al., 2022). However, our results indicated no association between muscle recovery and vitamin D status or 25OHD levels. This may be attributable to the fact that the majority of individuals presented vitamin D levels above the insufficiency threshold (>20.0 ng mL^−1^), so it would hardly be possible to detect potential associations even if they existed.

## 5 Conclusion

There is inter-individual variability, i.e., high and low responders, in the hamstring muscles response to a sport-specific RST, identifiable by MVIC torque assessment through the *90°*_*hip*_ *:20°*_*knee*_ *test*, which is a powerful indirect test to monitor hamstring muscle recovery in both women and men, in a cost-effective way. However, although female responders showed impaired muscle structure and function, the *Jurdan test* may not show such impairment in sportswomen after a sport-specific RST. Loss of muscle function is associated with impaired mitochondria, triggering an increase in oxidative stress status and circulating irisin in both HR females and males. Finally, muscle function remains unrecovered during the 72-h recovery period, revealing that 3 days will not be adequate time to restore hamstring structure and function after a sport-specific RST in both female and male responders.

### 5.1 Limitations

The study also presented some limitations to highlight when interpreting the results. First, while *a priori* computed sample size was sufficient to achieve the target statistical power, the number of participants was lowered when splitting into groups according to the *post hoc* analysis criteria of Paulsen et al. (2012). However, the use of this classification accounts for inter-individual variations, and provides a better presentation and interpretation of the response to exercise-induced residual fatigue (Paulsen et al., 2012). Finally, muscular and biochemical markers of residual fatigue were affected during the 72-h recovery period after the RST in HR individuals. Consequently, further research conducting additional blood and muscle function measurements, e.g., +96 h, until complete recovery are required to better understand the potential implications of RST on hamstring muscles recovery.

### 5.2 Perspectives

Two sprint and hamstring-specific muscle function tests (*90°*_*hip*_ *:20°*_*knee*_ *test* and *Jurdan test*) were examined to determine muscle recovery after sport-specific RST in women and men. Using this screening, health professionals and practitioners can better determine the hamstring recovery status of sportswomen and men, and thus more accurately individualize training programs in multiple sprint-based sports in order to reduce injury risk. Furthermore, the findings addressed new areas of applied research, using an integrative-based and cost-effective method to monitor exercise-induced OS, and suggesting irisin as a key link between exercised-induced MD/OS and muscle recovery. Nevertheless, it remains unclear whether (i) other types of exercise training, e.g., resistance training, or (ii) the sport practice *per se*, e.g., football match, influence the relationship between hamstring-specific muscle function and the recovery kinematics of MD, OS and/or irisin.

## Data Availability

The raw data supporting the conclusion of this article will be made available by the authors, without undue reservation.

## Abbreviations

25OHD, 25-hydroxyvitamin D; A.max, peak acceleration; AMPK, AMP-activated protein kinase; Ca^2+^, calcium ion; CK, creatine kinase; CV, coefficient of variability; F0.rel, relative (to body mass) theoretical maximal horizontal force production; FGC, force-generating capacity; FNDC5, fibronectin domain-containing 5 protein; HR, high-responders; IL, interleukin; K.fitted, rate of force development normalized by peak force; LR, low-responders; MD, muscle damage; MDA, malondialdehyde; MVIC, maximal voluntary isometric contraction; NF-κB, nuclear factor kappalight-chain-enhancer of activated B cells; OS, oxidative stress; OSI, oxidative stress index; PGC-1α, peroxisome proliferator-activated receptor γ co-activator 1α; P.max.rel, relative peak power; ROM, range of motion; ROS, reactive oxygen species; RST, repeated-sprint training; sMtCK, sarcomeric mitochondrial CK; TAS, total antioxidant status; Time.P.max, time to peak power; TOS, total oxidant status; V0, theoretical maximal running velocity.
